# The Open Microscopy Environment (OME) Data Model and XML file: open tools for informatics and quantitative analysis in biological imaging

**DOI:** 10.1186/gb-2005-6-5-r47

**Published:** 2005-05-03

**Authors:** Ilya G Goldberg, Chris Allan, Jean-Marie Burel, Doug Creager, Andrea Falconi, Harry Hochheiser, Josiah Johnston, Jeff Mellen, Peter K Sorger, Jason R Swedlow

**Affiliations:** 1 Image Informatics and Computational Biology Unit, Laboratory of Genetics National Institute on Aging, National Institutes of Health, 333 Cassell Drive, Baltimore, MD 21224, USA; 2 Division of Gene Regulation and Expression, University of Dundee, Dow Street, Dundee DD1 5EH, Scotland, UK; 3 Department of Biology, Massachusetts Institute of Technology, 77 Massachusetts Avenue, Cambridge, MA 02139, USA

## Abstract

The Open Microscopy Environment (OME) defines a data model and a software implementation to serve as an informatics framework for imaging in biological microscopy experiments, including representation of acquisition parameters, annotations and image analysis results. OME is designed to support high-content cell-based screening as well as traditional image analysis applications. The OME Data Model, expressed in Extensible Markup Language (XML) and realized in a traditional database, is both extensible and self-describing, allowing it to meet emerging imaging and analysis needs.

## Abstract 2

The Open Microscopy Environment (OME) defines a data model and software implementation to serve as an informatics framework for imaging in biological microscopy experiments.

## Rationale

Biological microscopy has always required an 'imaging' capability: traditionally, the image of a sample was drawn on paper, or with the advent of light-sensitive film, recorded on media that conveniently allowed reproduction. The advent of digital detectors in microscopy has progressively expanded imaging capacity, transforming the biological microscope into an assay device that linearly measures the flux of light at different points in a cell or tissue. Almost all the vast clinical and research applications of digital imaging microscopy treat the recorded microscope image as a quantitative measurement. This is especially true for fluorescence or bioluminescence, where the signal recorded at any point in the sample gives a direct measure of the number of target molecules in the sample [1-4]. Numerical analytic methods extract information from quantitative image data that cannot be gleaned by simple inspection [5-7]. Growing interest in high-throughput cell-based screening of small molecule, RNAi, and expression libraries (high-content screening) has highlighted the large volume of data these methods generate and the requirement for informatics tools for biological images [8-10].

In its most basic form, an image-informatics system must accurately store image data obtained from microscopes with a wide range of imaging modes and capabilities, along with accessory information (termed metadata) that describe the experiment, the acquisition system, and basic information about the user, experimenter, date, and so on [11,12]. At first glance, it might appear that these requirements can be met by applying some of the tools that underpin modern biology, such as the informatics approaches developed for genomics. However, it is worth comparing a genome-sequencing experiment to a cellular imaging experiment. In genomics, knowledge of the type of automated sequencer that was used to determine the DNA sequence ATGGAC... is not necessary to interpret the sequence. Moreover, the result ATGGAC... is deterministic - no further analysis is required to 'know' the sequence, and in general, the same result will be obtained from other samples from the same organism. By contrast, an image of a cell can only be understood if we know what type of cell it is, how it has been grown and prepared for imaging, which stains or fluorescent tags have been used to label subcellular structures, and the imaging methodology that was used to record it. For image processing, knowledge of the optical transfer function, spectral properties and noise characteristics of the microscope are all critical. Interpretation of results from image analysis requires knowledge of the precise characteristics of the algorithms used to extract quantitative information from images. Indeed, deriving information from images is completely dependent on contextual information that may vary from experiment to experiment. These requirements are not met by traditional genomics tools and thus demand a new kind of bioinformatics focused on experimental metadata and analytic results.

In the absence of integrated solutions to image data management, it has become standard practice to migrate large amounts of data through multiple file formats as different analysis or visualization methods are employed. Moreover, while some commercial microscope image formats record system configuration parameters, this information is always lost during file format conversion or data migration. Once an analysis is carried out, the results are usually exported to a spreadsheet program like Microsoft Excel for further calculations or graphing. The connections between the results of image analyses, a graphical output, the original image data and any intermediate steps are lost, so that it is impossible to systematically dissect or query all the elements of the data analysis chain. Finally, the data model used in any imaging system varies from site to site, depending on the local experimental and acquisition system. It can also change over time, as new acquisition systems, imaging technologies, or even new assays are developed. The development and application of new imaging techniques and analytic tools will only accelerate, but the requirement for coherent data management and adaptability of the data model remain unsolved. It is clear that a new approach to data management for digital imaging is necessary.

It might be possible to address these problems using a single image data standard or a central data repository. However, a single data format specified by a standards body breaks the requirement for local extensibility and would therefore be ignored. A central image data depository that stores sets of images related to specific publications has been proposed [13,14], but this cannot happen without adaptable data management systems in each lab or facility. The only viable approach is the provision of a standardized data model that supports local extensibility. Local instances of the data model that store site-specific data and manage access to it must be provided along with a mechanism for data sharing or migration between sites. These requirements are shared by other data-intensive methodologies (for example, mass spectrometry and two-dimensional gel electrophoresis). Thus, a major challenge is the design and implementation of a system for multidimensional images, experimental metadata, and analytical results that are commonly generated in biological microscopy that will also be generally adaptable to many different types of data.

To make it possible to manipulate and share image data as readily as genomic data, we are building an image-management system geared to the specific needs of quantitative microscopy. The major focus of the Open Microscopy Environment (OME) [11,15] is not on creating image-analysis algorithms, but rather on the development of software and protocols that allow image data from any microscope to be stored, shared and transformed without loss of image data or information about the experimental setting, the imaging system or the processing software. OME provides a data model that can integrate with other efforts to define experimental, genomic, and biological ontologies [16-19] and that is suitable for traditional low-volume microscopy and for high-throughput image-based screening. This data model is implemented in a relational database and application server to import, store, process, view and export data. The OME Data Model is also implemented in an Extensible Markup Language (XML) file format that makes it possible to transfer OME files between OME databases and exchange them with other software, including that provided by commercial vendors. OME does not replace or compete with existing commercial software for controlling microscopes, acquiring images or performing image restoration. Instead, it serves as a neutral broker among a multitude of otherwise incompatible software tools.

In our previous work [11], we described the conceptual foundation for an image informatics system. In this report we describe the implementation of this system, including details of the OME XML file format, a description of how images are represented both in the file format and in the data model, the application of semantic types for metadata extensibility as well as their use in modular image analysis, and describe recently developed software that makes use of this system and is targeted at end-users. The current version of OME focuses on fluorescence microscopy, but the underlying schema and file specifications can be extended to support any type of microscope image. The OME XML file format has already gained acceptance within the microscopy community. At the time of writing, two companies support the format in their current commercial offerings (Applied Precision, Issaquah, WA and Bitplane, Zurich, Switzerland), and it has been proposed as a standard recommendation for image data migration by the European Advanced Microscopy Network [20]. Immediate applications for OME within biomedical research include the characterization of dynamic cell and tissue structures for basic research, high-content cell-based screening and high-performance clinical microscopy.

## Definition of an image

**Figure 1:**
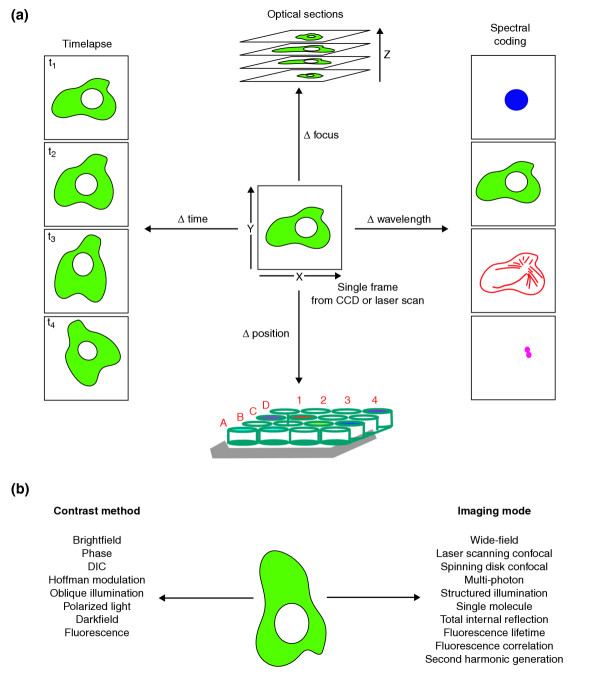
The mode of acquisition defines the pixel image data. The meaning of a 2D-image recorded from a digital microscope imaging system varies depending on how it is collected. Almost all of the different modes in (a) and (b) can be combined to analyze cell structure and behavior. All of the parameters and configurations must be somehow recorded for the interpretation of the pixel data in an image. **(a) **The spatial, spectral and temporal context of an image is used to generate more information about the cell under study. Changing stage position, focus, spectral range or time of imaging all expand the meaning of an image. Modified from [33]. **(b) **The two aspects of the image data collection that define the pixel data. A variety of methods are used to generate contrast in modern biological imaging. In addition, the imaging method used to record the data also has meaning.

All imaging experiments occur within specific temporal and spatial limits. In OME, we define an image as a five-dimensional (5D) structure containing multiple two-dimensional (2D) frames (Figure 1a). Each frame has dimensions (*x*, *y*) that correspond to the image plane of the microscope and is recorded from an array detector (for example a CCD camera in a wide-field microscope) or generated by a two-dimensional raster scan (for example, a laser scanning confocal microscope). Each frame has a specified focal position *z*, a wavelength, or more generally channel, *c*, and timepoint *t*. The extent of a 5D-image is unlimited. The time and channel dimensions may be continuous or discrete. For example, the image may contain an entire spectrum at each pixel as in Fourier Transform Infrared (FTIR) imaging, or it may consist of a set of discrete wavelengths such as commonly seen in fluorescence microscopy. Similarly, there may be a continuous series of time points that are evenly spaced, as in a video stream, or the image may contain unevenly spaced, discrete time points. Images that are not continuous in space are treated as separate images even though they may be part of the same experiment. For example, visiting several places on a microscope slide or a microtiter plate will result in as many separate images. Finally, the meaning of the pixel values recorded in each frame are determined by the imaging method performed (Figure 1b).

## The OME Data Model

To solve the problems of data interoperability and extensibility, we have developed a definition, or ontology, of the different data types and relationships included in an imaging experiment. The OME Data Model integrates binary image data and all information regarding the image acquisition and processing, and any results generated during analysis. In this way, all aspects of the data acquisition, processing, and analysis remain linked and can be used by any analysis or visualization application. Groups of Images can be organized into 'Datasets' and 'Projects'. (Throughout this paper, when referring specifically to OME objects (such as Projects, Datasets, Images, Pixels, and Features), they are capitalized.) Datasets are user-defined groups of images that are always analyzed together: an example would be images from a single immunofluorescence experiment. An image may belong to one or more datasets. Projects in turn are collections of datasets, and any given dataset may belong to one or more projects. Each project and dataset has its own name, description and owner.

The OME Data Model allows for other types of image collections. Explicit support is included for high-content assays (HCAs) conducted on microtiter plates or other arraying formats. In this case, the OME Data Model allows for an additional grouping hierarchy: 'Plates', 'Screens', 'Wells', and 'Samples'. Samples are groups of images from one well, Plates are groups of Wells, and Screens are groups of Plates. Just like Projects and Datasets, each level of the hierarchy has its own set of identifiers. It is also possible for a given plate to belong to multiple screens, thereby providing a logical mechanism for reuse of the same collection of data for different analyses. Similarly, a mechanism is provided for categorizing images into arbitrary user-defined groups.

An additional level of hierarchy below images included in the OME Data Model is 'Features'. Although there is some conflict of nomenclature in what is considered an image feature between areas of machine learning and traditional image analysis, in OME's case, image features are 'regions' in an image (for example cells or nuclei). Numerical descriptors used for classification content are then referred to as 'Signatures' [21]. The OME Data Model allows features to contain other features, so that, for example, the relationship between a cell, a nucleus and a nucleolus can be expressed. At present, we do not specify an ontology for the kinds of information an image feature may contain. Any information obtained by segmentation algorithms, or other algorithms that define Features is stored using the data model's extensibility mechanism (see Semantic types below).

### Semantic types

All information in the OME Data Model can be reduced to 'semantic types' (STs). In most ways, this is merely a name or label given to a piece of information, but in OME it has additional consequences. STs can describe information at four levels in the OME hierarchy: Global, Dataset, Image and Feature. Global STs are used to describe 'Experimenters', 'Groups', 'Microscopes', and so on - items that are applicable to all images in an OME database. Dataset STs are used to describe information about datasets - information pertinent to a collection of images. Image STs describe information pertinent to images, and feature STs describe information about image features - objects or 'blobs' within images. In our nomenclature, the data type is an ST, and the data itself is an attribute. For example, the 'Pixels' data type is an Image ST, and a particular set of Pixels is an attribute of a particular Image. Throughout this paper XML elements defined in the OME XML schema are placed within angle brackets (<>).

### Data model extensibility

Standardizing access to data solves many problems, but could severely limit the types of data that might be stored. Because it is not possible to define *a priori *what kinds of imaging experiments and analyses will be performed, it is not possible to design a data model to contain this information ahead of time. For this reason, we have included a mechanism for describing new types of data in the OME Data Model. As one of our goals is to define a common ontology for light microscopy, the STs that make up this ontology are part of the 'core set', whereas other STs can be locally defined to address evolving imaging needs. Since the data model contains its own description, it can be extended in arbitrary ways. As these extensions become commonly used, the STs that define them can be incorporated into the core set. The initial core set is concerned chiefly with acquisition parameters so that image data can be interpreted unambiguously. As the project evolves, analytical STs will be incorporated into the core set in order to achieve interoperability not only at the level of interpreting raw image data, but also at the level of interpreting image analysis results.

Consider an example where a commercial software vendor might specify additional metadata in the timing information for acquisition of Z sections in an XYZ 3D stack of image planes. As the timing information would pertain to specific images, this new data type would be declared as an Image ST. More specifically, since the timing information pertains to individual planes within the 5D Image, a set of plane indexes would be included in the definition referring to a specific plane. The timing information itself can be expressed as a delta-time or an absolute time (or both), and may have units that are either implied or made explicit. Regardless of how the timing is expressed, it is understood that any software that uses this newly declared ST agrees on the convention adopted and the precise meaning of the data it represents. This agreement on meaning allows any software application to exchange acquisition timing information with any other.

Using OME XML (see OME XML file below), this declaration would be stored in the <SemanticTypeDefinitions> element in the XML document, while the timing information itself (the attributes) would be stored under the <CustomAttributes> element for the specific image. The names of the elements under <CustomAttributes> match the names of the STs, and the data itself goes into the element's attributes. For example:

<CustomAttributes>

    <AcquisitionTiming theZ='0' theC='0' theT='0' deltaT='0.001'/>

</CustomAttributes>

Importantly, our open-source implementation of OME (see below) will automatically expand its database schema when it comes across an ST definition, and will populate the resulting tables when it comes across the data in <CustomAttributes>. This approach allows for immense flexibility in the ontologies OME can support.

### IDs and references

OME has adopted the Life Science ID (LSID) system of data registration [22]. Since LSIDs are universally unique, every piece of information stored using the OME Data Model can be traced to its source - regardless of how it was produced. Every OME element that has an ID attribute may follow the LSID format, but this is not a requirement. If a particular ID does not follow the LSID format (it does not start with 'urn:lsid:'), it must be assumed that this is a 'brand new' object. While this is a valid assumption for data, it may not be valid for an instrument description. For this reason actual globally unique LSIDs are preferred whenever possible - especially for global data (such as Experimenters, Screens, Plates, Microscopes). If the object is identified with a proper LSID, it can be referred to from other documents. In this way, a single document can be used to describe a microscope and its components, and subsequent documents containing images can refer to these components by LSID. There are open-source implementations of LSID servers (resolvers) and clients developed by IBM Life Sciences available online [22] that make it possible to resolve an LSID remotely. Although we plan to incorporate LSID resolution into OME software tools, at the time of writing, support for LSIDs are only incorporated into the OME Data Model.

The globally unique nature of LSIDs allows OME to trace every piece of information back to its origin. Provenance and data history will be discussed in a future report detailing the OME analysis system, but the use of LSIDs and a representation of data history is sufficient to determine the origin of every piece of information about an image. From precisely where, when and how the image was acquired, through any analysis that was done, to any structured information or conclusions that were derived as a result of analysis. LSIDs allow preservation of this chain of provenance regardless of the number of intermediate documents, and proprietary or open-source OME-compatible software systems that operated on this information.

## The OME XML file

The OME Data Model serves as the foundation of two tools we have developed to address the requirement for extensible image data management. The first addresses the absence of a universally recognized image data file format. We have built an XML-based implementation of the OME Data Model that can be used by manufacturers of acquisition hardware and developers of image-processing and analysis software who may not want to invent their own image format. With this definition, it is possible to specify a minimal set of commonly used parameters during image acquisition in light microscopy, analogous to the MIAME standard that defines a minimal set of information about microarray experiments [23].

**Figure 2:**
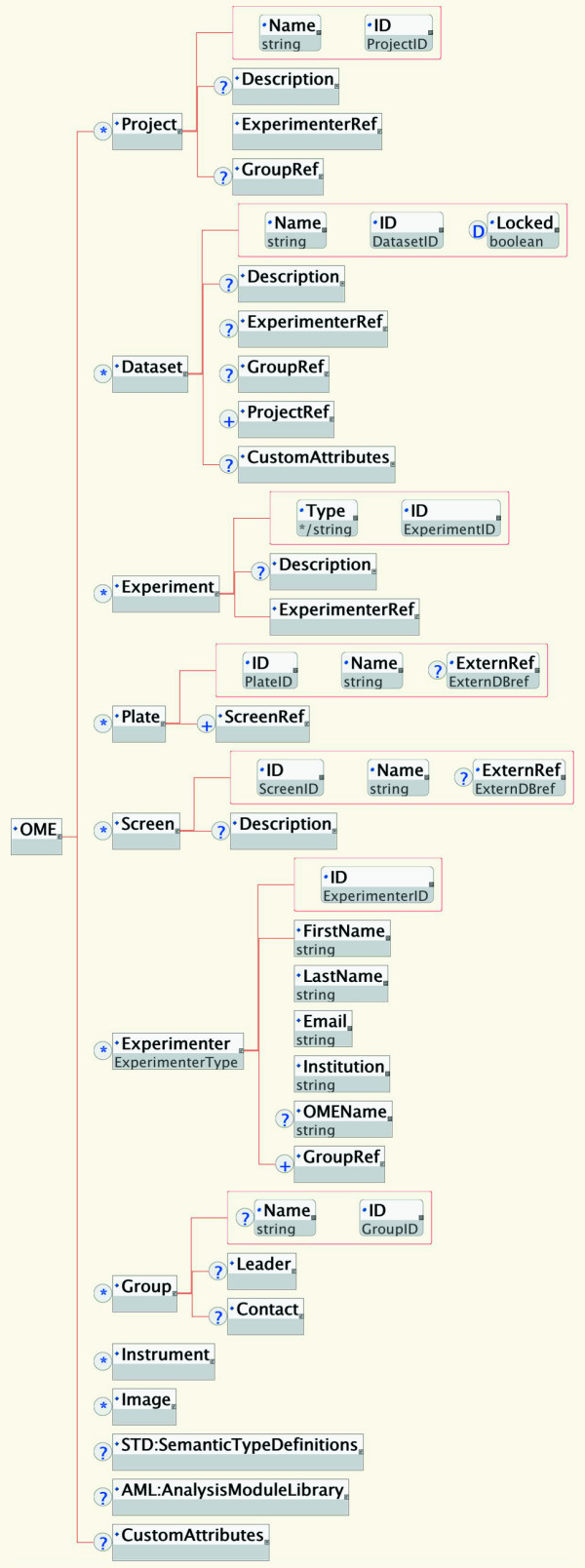
High-level view of the elements in the OME file schema. This figure (and Figures 3 and 4) should be read from left to right. A data type (for example, OME) is defined by a number of elements. In this case, OME is defined by Project, Dataset, Experiment, Image, and so on. Each of these elements can be defined by their own individual elements. The Image and Instrument elements are expanded in Figures 3 and 4. The full XML schema is available [24]. The full documentation for the schema is also available [25]. +, One or more elements of this type; ?, optional element or attribute; *, zero or more elements of this type; 1, choose one from a list of elements; D, the value of this element/attribute is constrained to one of several values, a range, or a text pattern (see the online documentation for more details [25]).

**Figure 3:**
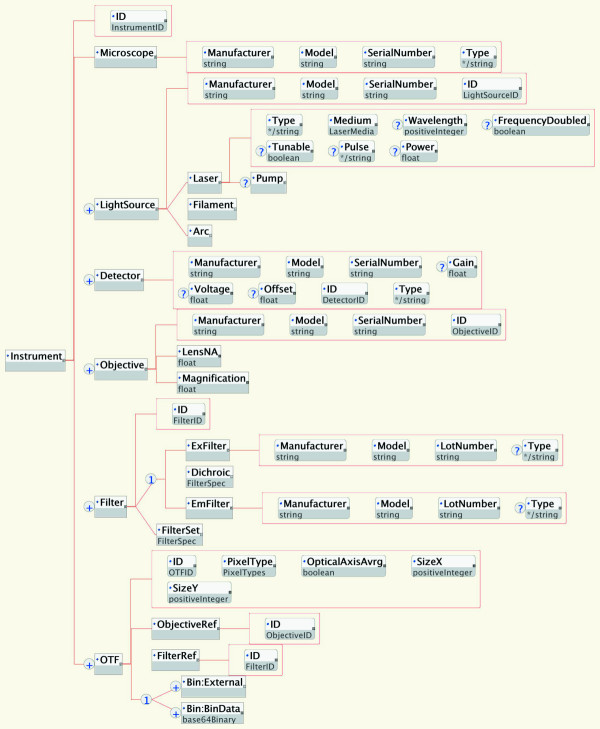
The Instrument element in the OME file schema. The data elements that define the acquisition system parameters are shown. For these descriptions, we have incorporated suggestions from many colleagues and commercial partners [32]. Symbols are as in Figure 2.

**Figure 4:**
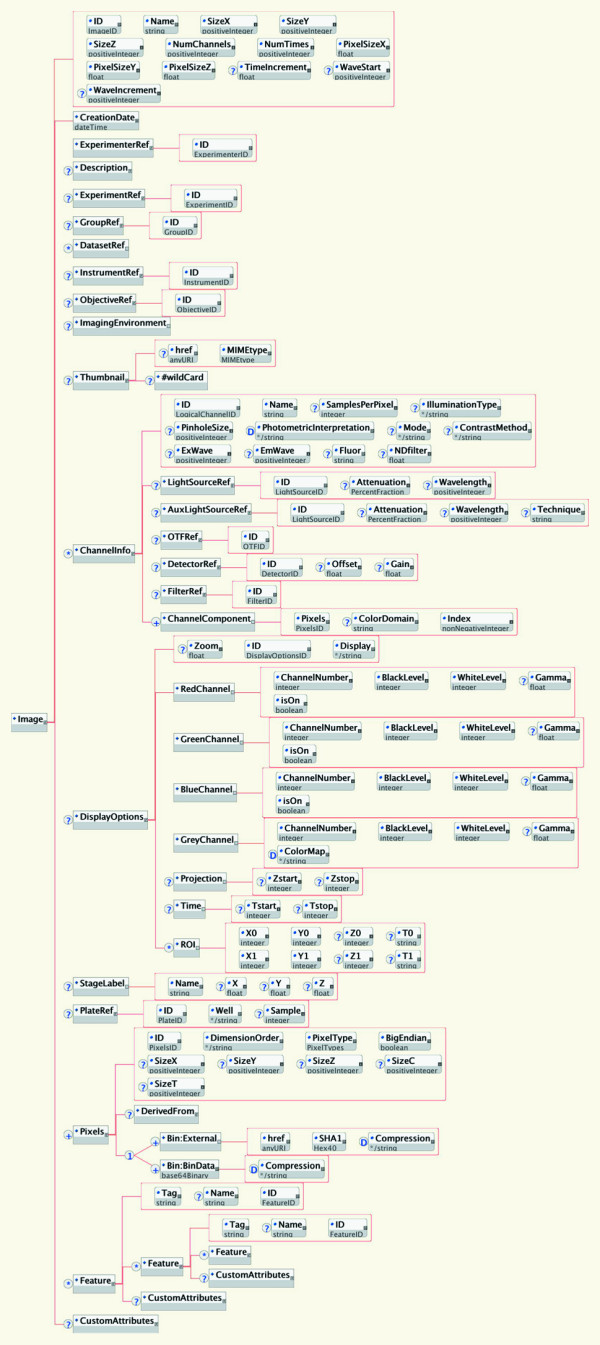
The Image element in the OME file schema. The data elements that define the an image in the OME file are shown. These include the image itself (Pixels), and a variety of characteristics of the image data and display parameters. Symbols are as in Figure 2.

All the characteristics of the OME Data Model described above are reproduced in the OME XML file. Along with each 5D image (that is, the binary pixels), the OME XML file contains all of the associated metadata. The OME file schema [24] and the full documentation for the schema [25] are available online. A description of how the schema is designed and its relationship to other OME schemas is also available online [26]. Figures 2, 3, 4 highlight some of the features of the schema. In these figures, the highest level in the schema is on the left side of the diagram, and the elements defined in it are read moving from left to right.

### Why XML?

The structure of the OME XML document is defined in XML Schema, which is a standard language for defining XML document structure [27]. The use of XML and a publicly available schema allows OME documents to be used in several ways that are not possible with current image formats. For example, modern browsers incorporate XML parsers, and are able to display the information contained in XML with the use of a style sheet, thus allowing customized display of data in the document using a standard browser without additional software. The use of XML also allows us to take advantage of its growing popularity in various unrelated fields - including a great deal of software written for XML, including databases, editing tools, and parsing libraries. Finally, and perhaps most important, XML is a plain-text format. As a last resort, it can be opened in any text editor and the information it contains can simply be read by a person. This inherent openness is one of its most desirable features for representing scientific data.

Defining the OME file using XML Schema allows other advantages. The document structure is specified in a form that can be parsed, which allows third-party software to validate XML documents against our published schema. This formal specification allows other parties to implement this format without the potential misunderstanding and incompatibility that is common with textual descriptions of file formats. For example, several manufacturers are either developing or have developed support for the OME file format independently of each other and, to a large extent, independently of our group of developers. No exchange of intellectual property or reverse engineering is necessary to accomplish this. The XML Schema is the definitive documentation for reading and writing OME XML files, used in the same way by third-party developers for proprietary software, as well as by ourselves for our own open-source implementation.

There are a few disadvantages to XML worth considering. A commonly perceived weakness of XML is that its human-readable design is often at odds with the storage of binary data. Since the bulk of an image file is represented by the pixels in the image and not the metadata, this might be perceived as a serious problem. A related problem is that XML is verbose - XML files are often much larger than their binary equivalents, and image files are already quite large. The proposed format addresses these two concerns by storing binary data in plain text and reducing file size using compression.

The standard approach to representing binary data in XML is with the use of base64 encoding. A 24-digit base 2 binary number (three bytes) is converted to a 4-digit base 64 number (four bytes) with each digit represented as a text character using all the numbers, upper- and lowercase letters and two punctuation marks. This conversion inflates the size of the binary data by 25%. To mitigate this increase in size, OME XML specifies compression of the pixels on a per-plane basis in either bzip2 or gzip, both patent-free compression schemes available in open-source form online. Owing to the high compressibility of image data, OME XML files are in practice much smaller than their equivalents in other formats, usually a half to a third the size of uncompressed binary data. Because the compressed stream is still encoded in base64, it still incurs the 25% overhead, but on a much smaller piece of binary data. Of course text is itself easily compressed, and the gzip format is a standard encoding for XML, so any XML software library will transparently read and write these compressed files even though the compressed file will no longer be readable by standard text editors. However, this secondary compression will only eliminate the base64 encoding overhead - it will not further compress already compressed planes.

There are limitations to the use of this compression scheme. Performing the compression on a per-plane basis allows limited random access to the planes. The entire XML file need not be kept in memory in order to access arbitrary planes by index, but a file offset cannot be calculated for a given plane due to their different sizes when compressed. Instead, the entire file has to be scanned first in order to determine the file offsets for each plane index. It is important to note that the primary goal of the OME XML file format is not raw performance, but interoperability above all else, using widely accepted standards and practices for information exchange. As the OME XML file format has gained acceptance, a demand for a high-performance variant has begun to emerge, and we are examining several possibilities that preserve the metadata structure that we have defined, but allow rapid reading and writing from disc.

## Schema overview

Figure 2 shows the main elements of the OME XML file schema. As discussed above, each image is defined as being part of a dataset and project, and when necessary, a given plate and screen. The stored data is also related to the experimenter that collected the data and his or her group. Any additional types of global data including customized or vendor-specific data can be defined at this level. Images and Instruments are defined as discussed below. Many of the elements contain IDs that uniquely identify that data element - Experimenter, Dataset. If these identifiers follow the LSID format they are considered globally unique and can be used as references between other OME XML documents or remote OME installations.

This format allows for an arbitrary number of images to be described and their relationships and grouping patterns specified in a single document. Conversely, the file may describe only the imaging equipment, users, or other parameters at a given site and not contain any images. Subsequent documents can refer to these items by LSID. Or, as is done in other formats, the file can be used to specify a single image and its accompanying metadata. As any information not specified in the schema must be represented as well, a section is dedicated to defining new types of information (<SemanticTypeDeclarations>). The information itself is specified at the appropriate hierarchy level within the <CustomAttributes> elements that exist in <OME>, <Dataset>, <Image> and <Feature>.

The least developed aspect of the OME schema is the Experiment description. Although clearly a critical part of the metadata, the design of this ontology is under development by many other groups (for example, MIAME/MAGE, Gene Ontology (GO), Proteomics Standards Initiative (PSI), and minimum information specification for *in situ *hybridization and immunohistochemistry experiments (MISFISHIE)) [16-19] and we are experimenting with several scenarios for merging these efforts with OME. At present, several of these projects including OME are evaluating the new Web Ontology Language (OWL) recommendation from the World Wide Web consortium (W3C) to standardize ontology specification for the Semantic Web initiative [28]. At the moment, Experiment is defined in simple unstructured text entered by the user. This situation reflects our goals of not only defining a data model or ontology, but also building the tools for using that model in demanding, experimentally relevant, data-intensive applications. However, it is worth noting that a separate group has represented the OME Data Model within the Resource Description Framework (RDF), and has begun using this implementation [29]. We are currently studying an implementation of OME in OWL, and whether an RDF-based system provides the performance required for large-scale imaging applications.

### The OME Instrument type

The OME Instrument type (Figure 3) provides a description of the data-acquisition instrument and defines the actual instrument as well as available configuration choices such as the objective lens, detector, and filter sets. Instrument also defines the use and configuration of lasers or arc lamps and includes a specification for a secondary illumination source (for example, a photoablation laser). Once defined in the Instrument, the specific components used to acquire an image (or a channel within an image) are referenced from within the Image or its ChannelInfo elements. The <Instrument> element is meant to define a static instrument composed of several components: one or more light sources, one or more detectors, filters, objectives, and so on. Because it does not change from image to image and has a globally unique LSID, it does not need to be defined in every OME file with images collected from it. The Image elements within the OME File contain references to the instrument's components along with any necessary parameters for their use (that is detector gain). The Instrument may also contain several optical transfer functions (OTFs), which can be referred to from the ChannelInfo element, allowing each channel within a set of pixels to specify its own OTF.

### The OME Image type

The OME Image type (Figure 4) provides a description of the structure, format, and display of the image data. There are references to the light source, spectral filtering, imaging method, and display settings used for each channel. The actual binary data, referred to as 'Pixels' are also stored in this part of the schema. A set of Pixels is a 5D-structure containing multiple 2D-frames collected across focus (*z*), wavelength or channel (*c*), and time (*t*), as described above. Sets of Pixels that are not continuous in space are treated as separate images even though they may be part of the same experiment.

The Image's binary pixels are compressed and encoded in base-64 as described above, with one plane per <BinData> element. The schema allows for more than one set of Pixels in an Image. A given image may consist of the original 'raw' pixels and a set of processed pixels as is often done for deconvolution or restoration microscopy. Because these two sets of pixels share the same acquisition metadata, they are grouped together in the same image.

A critical feature in this specification is a definition of what the data stored in 'Pixels' actually mean. The meaning of the pixels is stored as three attributes in <ChannelInfo>: Mode, ContrastMethod, and IlluminationType. Mode describes the microscopy method used to generate the pixels, and can take on values such as 'Wide-field', 'Laser-scanning confocal', and so on. ContrastMethod describes how contrast is developed in the type of microscopy used and can contain terms such as 'BrightField', 'DIC', or 'Fluorescence'. The IlluminationType attribute describes how the sample was illuminated and can contain values of 'Transmitted', 'Epifluorescence', and 'Oblique'. Together these terms and their controlled vocabulary describe how the pixels were acquired. Each <ChannelInfo> has several internal elements that allow further refinement of the acquisition parameters by referring to components defined in the <Instrument>, such as filters and light sources. Each channel in the image has its own <ChannelInfo>, allowing the description of multimode images.

The metadata associated with a channel have an additional important feature made possible with the nested <ChannelComponent> element. In a fluorescence experiment, each fluorescence channel would be described by a <ChannelInfo>, and each of these would contain a single <ChannelComponent> referring to an index in the *c *dimension of the Pixels. However, in several imaging modes, each channel may contain several components. For example, in fluorescence-lifetime imaging, each fluorescence channel may contain 128 bins of fluorescence-lifetime data. The image may consist of lifetime measurements for several fluorescence channels. In this case, each fluorescence channel would still be represented by a single <ChannelInfo>, but each of those would have 128 <ChannelComponent>s. This allows the channel dimension to effectively represent two dimensions - a logical channel containing all of the metadata and one or more components representing the actual data. The same mechanism can be used to represent data from FTIR imaging.

### Updating the OME file specification

The OME XML file has been developed with input from the OME consortium and a number of commercial partners (see Figure 3 legend). However, the specification for this format is incomplete and doubtless will be updated to accommodate unanticipated requirements. Moreover, as new data acquisitions methods develop, new data semantics and elements will be required. However, modifications to the specification for this file must occur in stages, preceded by announcements, if it is to be used as an export format. The OME file allows modifications to the schema to be implemented and tested through the Custom Attributes type. Proposed new types and elements can be tested and modified there, and then when fully worked out and agreed upon by the OME community, can then be merged into the main schema.

## The OME database

It is formally possible to use a library of OME XML files as a data warehouse. A true image informatics system however, must also maintain a record of all transactions with the data warehouse, including all data transformations and analyses. Storing and recording image data is a first step; a defined set of interfaces and access methods to the data must be also be provided. For this reason, we have developed a second implementation of the OME Data Model as a relational database that is accessed using a series of services and interfaces. All of these tools are open source and licensed under the GNU Lesser General Public License (LGPL) [30]. The initial design has been described previously [11] and a description of more recent updates is available [15]. Image metadata are captured by the OME database when it imports a recognized file format, and are then available either by accessing the database directly or through a variety of interfaces into the OME database. These will be the subject of a future publication, but source code and documentation are available [31]. An important consequence is that all commonly available types of metadata are stored in common tables. It is not necessary to know the format of the underlying file in order to access this information. For example, to find the exposure time for a particular image, one would look in the same table regardless of the commercial imaging system used to record the data.

The use of an OME database as a record of all data transformations contrasts with the standard approach to image processing. In a stand-alone analysis program, data relationships are specified by the programmer and are therefore 'hard-coded'. The results, while useful, do not usually link to the original data or other analyses. In an OME database, an identical algorithm can be used, but the resulting data are returned to the database, and are linked to the algorithm that produced them. A subsequent analysis can gather its inputs from the database as well, without having to link directly to the previous algorithm directly. The links between measurements, results and the image data can be incorporated into other analyses defined by the user. Trends and relationships between these can easily be tested. Most important, the complete transactional record of data elements is known and is available, in effect creating a transfer function for data analysis. This kind of data provenance for biological microscopy has sometimes resided in lab notebooks, sometimes coded in filenames, or sometimes simply retained only in experimenters' memories. With OME, it is finally stored, managed, and available in a generally accessible form.

**Figure 5:**
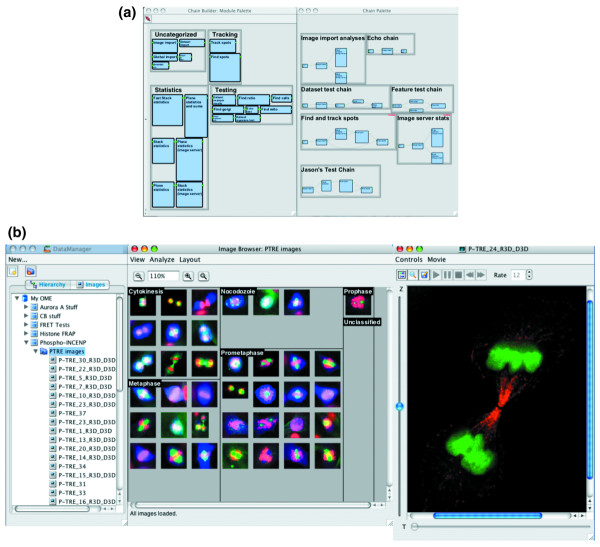
Using STs for visualization in OME. Examples of the use of STs for visualization of data within an OME database are shown. These tools are Java applications that access OME via the OME remote framework [34]. All OME code is available [31]. **(a) **The Chain Builder, a tool that enables a user to build analysis chains by ensuring that the input requirements of a given module are satisfied by outputs from previous modules. This is achieved by accessing the STs for the inputs and outputs within an OME database. **(b) **The DataManager, DatasetBrowser and 5DViewer. The DataManager shows the relationships between Projects, Datasets and Images within an OME database. The DatasetBrowser modifies the display method for images within a given dataset depending on the values of data stored as STs within an OME database. The 5Dviewer allows visualization of individual images based on STs in an OME database.

To function as planned, OME must ensure that requirements of different processing and analysis tools are satisfied before execution. To accomplish this, STs are used to govern what kinds of information can flow between analysis modules. In OME, analysis modules can exchange information only if the output of one has the same ST as the input of the next. This principle means that information can flow only between logically and semantically similar data types, not simply between numerically similar data types. This ensures that users employ algorithms in a logically consistent manner without necessarily an intimate knowledge of the algorithm itself. We have used this concept to implement a user tool called 'Chain Builder' (Figure 5a). This Java tool accesses the STs in an OME database and allows a user to 'chain' analysis modules together, linking of separate modules by matching the output STs of one module with the input STs of the next. Thus OME uses 'strong semantic typing', not only to store and maintain data and metadata, but also to define permitted workflows and potential data relationships.

Figure 5b shows a second example of the use of STs. In this example, a data manager (Figure 5b, left) displays the Projects, Datasets, and Images belonging to one OME user. Right-clicking a Dataset opens a Dataset browser (Figure 5b, middle) and displays image thumbnails obtained from the OME database. The browser accesses data associated with specific STs to define how an array of thumbnails should be presented to the user. In this case, the cell-cycle position of the cell in each image is used to define the layout (a more in-depth description of this tool is in preparation). Finally, a 5D-image viewer (Figure 5b, right) allows viewing of the individual images, with display parameters based on data obtained from an OME database associated with appropriate STs (signal min, max, mean, and so on).

### Data migration

Under most circumstances, the contents of a single OME database will be available only to the local lab or facility. However, data sharing and migration is often critical for collaborations or when investigators move to a new site. In OME, database export is achieved using the OME XML file. OME Images can be exported, along with their metadata, and analytic results and exposed to external software tools or imported to a second OME database. This strategy solves the file-format problem that has so far plagued digital microscopy.

### OME database extensibility

It is clear the OME Data Model, and its representation in a specific instance of an OME database will be adapted to support local experimental requirements. We have implemented this within the OME server code simply by loading an OME XML containing new STs and updating the existing database on the fly. However, an inherent problem in supporting schema extension is a potential for incompatibility between different schemas. If an OME database exports an OME XML file with a locally modified data model, how can that file be accessed by another OME site? Since OME defines what are considered core STs, all other STs must be defined within the same document that contains data pertaining to them. During import, local STs and imported STs are considered equal if their names, elements and element types are equal. In this way, if the structure of an ST can be agreed upon, the information it describes can be seamlessly integrated across different OME installations. If the structure of an extended ST is not agreed upon beforehand, then the STs are considered incompatible and their data are kept separate. If however, two STs have the same name, but different elements or element types, a name collision will result, and the import will be rejected until the discrepancy is resolved. Because the agreed on meaning and structure of STs is essentially a social contract and are not defined more formally, these name collisions must be resolved manually. A common approach to resolve name collisions is the use of namespaces - essentially a prefix to differentiate similar names from different schemas. While namespaces solve the immediate problem of collision, they do not address the underlying problem - that ST names and their meanings have not been agreed on. The disadvantage of using namespaces is they would not allow the information in these STs to be used interchangeably, and it is this interoperability rather than mere coexistence that is the desired result.

## Discussion

We have designed and built OME as a data storage, management and analysis system for biological microscopy. The data model used by OME is represented in two distinct ways: a set of open-source software tools that use a relational database for information storage, and an XML-based file format used for transmission of this information and storage outside of databases. The OME XML file format allows the exchange of highly structured information between independently developed imaging systems, which we believe is a major hurdle in microscopy today. The XML schema provides support for image data, experimental and image metadata, and any generated analytic results. The use of a self-describing XML schema allows this format to satisfy local requirements and enables a strategy for updating schemas to satisfy new, incoming data types. This approach provides the infrastructure to support systematic quantitative image analysis, and satisfies an indispensable need as high-throughput imaging gains wider acceptance as an assay system for functional genomic assays.

Our implementation of a relational database for digital microscopy satisfies the absolute requirement for local extensibility of data models. We acknowledge the impossibility of defining a single standard that encompasses all biological microscope image data. However, using the self-describing OME XML file, we can mediate between different data models, and when necessary, update a local model so that it can send or receive data from a different model. In this way, OME considers data dialects as a compromise between a universal data language and a universe of separate languages. In general, although the current OME system is focused on biological microscopy, its concepts, and much of its architecture, can be adapted to any data-intensive activity.
